# Astrocytic SARM1 promotes neuroinflammation and axonal demyelination in experimental autoimmune encephalomyelitis through inhibiting GDNF signaling

**DOI:** 10.1038/s41419-022-05202-z

**Published:** 2022-09-02

**Authors:** Lingting Jin, Jingjing Zhang, Xin Hua, Xingxing Xu, Jia Li, Jiaojiao Wang, Mianxian Wang, Huitao Liu, Haoyu Qiu, Man Chen, Xu Zhang, Ying Wang, Zhihui Huang

**Affiliations:** 1 Department of Neurology, The First Affiliated Hospital of Wenzhou Medical University, Wenzhou, Zhejiang China; 2 School of Basic Medical Sciences, Wenzhou Medical University, Wenzhou, 325035 Zhejiang China; 3 School of Pharmacy, and Department of Neurosurgery of the Affiliated Hospital,, Hangzhou Normal University, Hangzhou, 311121 Zhejiang China; 4 Clinical Research Center, Affiliated Hangzhou First People’s Hospital, Zhejiang University School of Medicine, Hangzhou, Zhejiang 310003 China

**Keywords:** Astrocyte, Neuroimmunology

## Abstract

Astrocytes are important components of the innate immune response in the central nervous system (CNS), involving in the inflammatory and neurotoxic responses that occur in CNS diseases, such as multiple sclerosis (MS). Recent studies have shown that SARM1 plays a critical role in axonal degeneration and inflammation. However, the detailed role of astrocytic SARM1 in MS remains unclear. Here, we established the MS model of mice - experimental autoimmune encephalomyelitis (EAE) and found that SARM1 was upregulated in astrocytes of the spinal cords of EAE mice. Moreover, conditional knockout of astrocytic *SARM1* (*SARM1*^*GFAP*^-CKO mice, *SARM1*^*Aldh1L1*^-CKO mice) delayed EAE with later onset, alleviated the inflammatory infiltration, and inhibited the demyelination and neuronal death. Mechanically, RNA-seq revealed that the expression of glial-derived neurotrophic factor (GDNF) was upregulated in *SARM1*^*−/−*^ astrocytes. Western blot and immunostaining further confirmed the upregulation of GDNF in spinal cord astrocytes of *SARM1*^*GFAP*^-CKO EAE mice. Interestingly, the downregulation of GDNF by streptozotocin (STZ, a drug used to downregulate GDNF) treatment worsened the deficits of *SARM1*^*GFAP*^-CKO EAE mice. These findings identify that astrocytic SARM1 promotes neuroinflammation and axonal demyelination in EAE by inhibiting the expression of GDNF, reveal the novel role of SARM1/GDNF signaling in EAE, and provide new therapeutic ideas for the treatment of MS.

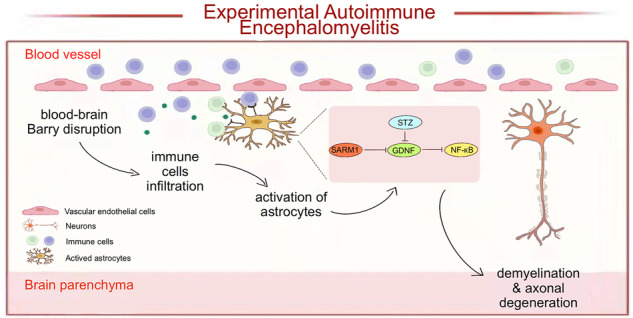

## Introduction

Multiple sclerosis (MS) is one of the autoimmune diseases whose clinical symptoms are monocular vision loss caused by optic neuritis, limb weakness or sensory loss caused by rhabdomyositis, and ataxia caused by cerebellar lesions, which seriously endangers the health of young people, especially women [1–4]. According to the latest diagnostic criteria, MS is divided into four subtypes: 1) clinically isolated syndrome (CIS); 2) relapsing-remitting (RRMS); 3) secondary progression (SPMS); 4) primary progression (PPMS) [4, 5]. The most common type is RRMS on which research of clinical drugs has achieved great progress nowadays. However, some patients can develop from RRMS to SPMS, and a small number of patients present with PPMS at first, which represents different pathological mechanisms among different MS types [6]. Therefore, according to the pathogenesis of different MS types, taking the corresponding treatment measures becomes the focus of researches.

Previous opinion thought that peripheral immune cells are activated to infiltrate the central nervous system (CNS), where an inflammatory cascade occurs to attack myelin components, resulting in axonal damage [7, 8]. However, some phenomena, such as loss of oligodendrocytes and myelin in the absence of T or B cell infiltration in early lesions [9]; extensive loss of myelin in the cerebral cortex and deep gray matter but little infiltrating immune cells [10–12] refute this theory. There is a growing awareness that peripheral immune cell-mediated inflammatory mechanisms can not fully explain the degenerative process. In fact, recent studies suggest that in progressive disease, the innate immune response of resident CNS cells may play a key role in myelin damage and axonal degeneration, especially the immunity of astrocytes and microglia. Astrocytes, the most abundant cell type in the CNS, play a pivotal role in MS [13]. It is a key component of the blood-brain barrier (BBB) function and interacts with BBB endothelial cells, pericytes and neighboring neurons, which controls the permeability of BBB [14]. In MS/EAE, astrocytes are activated. On the one hand, astrocytes secrete chemokines to recruit peripheral leukocytes into the CNS by disrupting the BBB, thereby causing an inflammatory cascade to damage myelin. Moreover, mitochondria and neurons are damaged by astrocytes by reducing neurotrophic factors and increasing NO, glutamate and other active substances [15]. On the other hand, leukocytes are restricted from entering the CNS because astrocytes participate in forming the glial scars [16]. Moreover, in acute and chronic injury, reactive astrocytes play a major role in defending against oxidative stress. Because of the powerful and diverse functions of astrocytes in MS, therapeutic approaches targeting them are expected to have a significant clinical impact on progressive disease.

In addition to inflammation and demyelination, axon damage is also an important part of MS, especially in the progressive stage of degeneration [13, 17, 18]. The wallerian degeneration (WD) was characterized by cytoskeletal granule disintegration, mitochondrial swelling, and axonal fragmentation. It is a classic model of axonal degeneration [19]. A recent study has shown that SARM1 (sterile alpha and TIR motif containing 1) induces activation of part of the molecular pathway for WD [20]. SARM1 is an intracellular protein that is highly expressed in the nervous system of mammals. It contains a C-terminal Toll/interleukin-1 receptor (TIR) domain, two tandem sterile alpha motif (SAM) domains and an N-terminal region with multiple armadillos repeat motifs (ARMs) [20, 21]. SARM1 is activated because of the reduction of nicotinamide mononucleotide adenylyl transferase (NMNAT2), which leads to the decrease of NADase activity in its TIR domain, NAD depletion, NMN accumulation, ATP energy depletion, Ca^2+^ influx increase, and finally axon degeneration [21, 22]. Interestingly, previous studies have shown that the effect of the inactivation of SARM1 on the immune response depends on the disease model or species. In human peripheral blood leukocytes, the function of SARM1 is to suppress the immune response [23–25]. However, in the animal models of CNS infection, the loss of SARM1 leads to a reduction in CNS inflammation, indicating that the function of SARM1 is to enhance the antiviral response of the CNS [26, 27]. In addition, it has been reported that SARM1 gene knockout not only can prevent neuronal degeneration and perinatal death but also can inhibit toxic neuropathy, such as amyotrophic lateral sclerosis (ALS) and glaucoma-related degeneration [28]. At present, the mechanism and pathway of SARM1 in neurons are relatively clear. In neurons, the activation of SARM1 stimulates the production of inflammatory cytokines and chemokines, and SARM1 stimulates the Jnk-c-Jun axis through the MAPK pathway, thereby initiating neuroinflammation responses [29]. However, it remains unclear what role the astrocytic SARM1 may play in MS and EAE.

In this study, we found that SARM1 was upregulated in the astrocytes of the spinal cords of EAE mice, and the knockout of astrocytic SARM1 alleviated neuroinflammation, inhibited demyelination and axon damage, and decreased the neuronal death in EAE mice. In addition, through RNA sequencing, we found that the expression of GDNF was upregulated in *SARM1*^*-/-*^ astrocytes. Moreover, the level of GDNF protein was significantly increased in *SARM1*^*GFAP*^-CKO EAE mice, compared with that in *SARM1*^*f/f*^ EAE mice. Since streptozotocin (STZ) is usually used to inhibit the expression of GDNF [30, 31], its treatment deteriorated the deficits of *SARM1*^*GFAP*^-CKO EAE mice, suggesting that astrocytic SARM1 may promote EAE through inhibiting GDNF signaling. Our findings provided some evidence for the mechanisms of demyelination and neuroinflammation in EAE, which may contribute to developing new MS therapies.

## Results

### SARM1 was upregulated in astrocytes of spinal cords of EAE mice

**Fig. 1:**
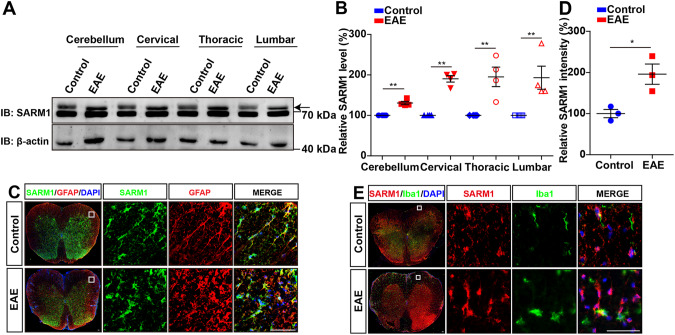
SARM1 was upregulated in astrocytes of spinal cords of EAE mice. **A** Western blot analysis of SARM1 expression in the cerebellum, cervical, thoracic and lumbar spinal cords of control and EAE mice. The higher molecular weight band is marked with an arrow as the band corresponding to SARM1. **B** Quantitative analysis of the relative SARM1 levels as shown in **A** (normalized to control mice, *n* = 4). **C** Double immunostaining analysis of SARM1 (green) and GFAP (red) in control and EAE mice. **D** Quantitative analysis of intensity of SARM1 **C** (*n* = 3). **E** Double immunostaining analysis of SARM1 (red) and Iba1 (green) in the spinal cords of control mice and EAE mice. Scale bar, 50 μm. Data were mean ± SEM. Student’s t-test, *p < 0.05, **p < 0.01.

To explore whether SARM1 is activated and the potential role of glial SARM1 in MS, we first examined the expression pattern of SARM1 in EAE mice. Western blot showed that the expression of SARM1 was significantly upregulated in the cerebellum, cervical, thoracic, and lumbar spinal cords of EAE mice (Fig. 1A, B). Further double immunostaining of SARM1 and glial markers, including GFAP (astrocytes marker) and Iba1 (microglia marker) showed that SARM1 was mainly detected in GFAP^+^ astrocytes (Fig. 1C, D), but not in Iba1^+^ microglia (Fig. 1E), which was consistent with previous research [20]. Moreover, the intensity of SARM1 in EAE mice was higher than that in the control mice (Fig. 1C, D), suggesting that SARM1 was upregulated in astrocytes of EAE mice.

### EAE was relieved with later onset, less inflammatory infiltration, and fewer neuronal death in *SARM1*^*GFAP*^-CKO mice

To further study the role of astrocytic SARM1 in EAE model, the floxed *SARM1* allele (*SARM1*^*f/f*^) mice were crossed with *GFAP-Cre* transgenic mice to generate *SARM1*^*GFAP*^-CKO mice. Indeed, SARM1 was effectively knockout in the cerebellum, cervical, thoracic and lumbar spinal cords of *SARM1*^*GFAP*^-CKO mice (Supplementary Fig. S1A, B), which indicated that *SARM1*^*GFAP*^-CKO mice were successfully constructed.

**Fig. 2:**
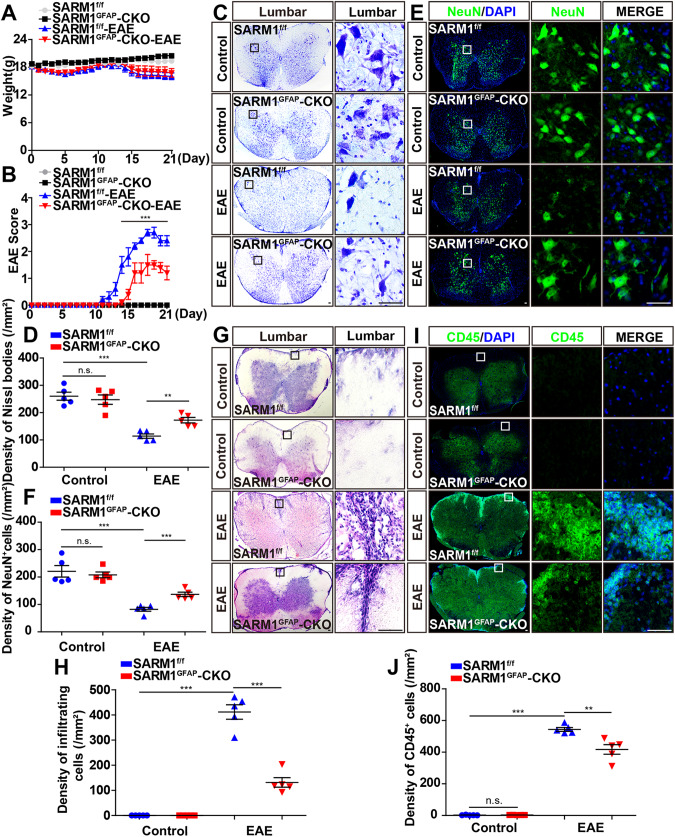
EAE was relieved with later onset, less inflammatory infiltration, and fewer neuronal death in *SARM1*^*GFAP*^-CKO mice. **A** The weight of *SARM1*^*f/f*^ mice and *SARM1*^*GFAP*^-CKO mice, and *SARM1*^*f/f*^ EAE mice and *SARM1*^*GFAP*^-CKO EAE mice ranged from 0 to 21 dpi (*n* = 5, two-way ANOVA with Bonferroni’s post-tests). **B** The EAE score of *SARM1*^*f/f*^ and *SARM1*^*GFAP*^-CKO mice ranged from 0 to 21 dpi (*n* = 5, two-way ANOVA with Bonferroni’s post-tests). **C** Typical images of Nissl staining in the lumbar spinal cords of *SARM1*^*f/f*^ mice and *SARM1*^*GFAP*^-CKO mice, and *SARM1*^*f/f*^ EAE mice and *SARM1*^*GFAP*^-CKO EAE mice. **D** Quantitative analysis of density of Nissl bodies as shown in **C** (*n* = 5). **E** Typical images of NeuN^+^ immunostaining in the lumbar spinal cords of *SARM1*^*f/f*^ and *SARM1*^*GFAP*^-CKO mice, and *SARM1*^*f/f*^ EAE and *SARM1*^*GFAP*^-CKO EAE mice. **F** Quantitative analysis of the density of NeuN^+^ cells as shown in **E** (*n* = 5). **G** Typical images of HE staining in the lumbar spinal cords in of *SARM1*^*f/f*^ mice and *SARM1*^*GFAP*^-CKO mice, and *SARM1*^*f/f*^ EAE mice and *SARM1*^*GFAP*^-CKO EAE mice. **H** Quantitative analysis of the density of infiltrating cells as shown in **G** (*n* = 5). **I** The typical images of CD45^+^ immunostaining in the lumbar spinal cords of *SARM1*^*f/f*^ mice and *SARM1*^*GFAP*^-CKO mice, and *SARM1*^*f/f*^ EAE mice and *SARM1*^*GFAP*^-CKO EAE mice. **J** Quantitative analysis of the density of CD45 ^+^ cells as shown in (**I**) (*n* = 5). Scale bar, 50 μm. The data were mean ± SEM. Student’s t-test unless otherwise indicated, n.s., not significant (*p* > 0.05), **p < 0.01, ***p < 0.001.

Next, female *SARM1*^*f/f*^ mice and *SARM1*^*GFAP*^-CKO mice aged 8–12 weeks were subjected to treatment with MOG_35–55_ and pertussis toxin to induce EAE. There was no significant difference in body weight between *SARM1*^*f/f*^ EAE mice and *SARM1*^*GFAP*^-CKO EAE mice, but the EAE score of *SARM1*^*GFAP*^-CKO mice was significantly reduced and the peak time of onset was delayed, suggesting that dyskinesia of EAE was alleviated in *SARM1*^*GFAP*^-CKO mice (Fig. 2A, B).

Further Nissl staining revealed that there was no difference in the density of Nissl bodies in the control group, suggesting that *SARM1* knockout in astrocytes had no effect on the development of spinal cords (Fig. 2C, D). In *SARM1*^*f/f*^ EAE mice, the density of Nissl bodies was significantly decreased, compared with that in the control *SARM1*^*f/f*^ mice, however, the loss of Nissl bodies was significantly reduced in *SARM1*^*GFAP*^-CKO EAE mice (Fig. 2C, D). Furthermore, immunostaining also showed the loss of NeuN^+^ cells was significantly decreased in the lumbar spinal cords of *SARM1*^*GFAP*^-CKO EAE mice, compared with that in *SARM1*^*f/f*^ EAE mice, further confirming that the neuroprotective effect of deletion of astrocytic *SARM1* in EAE model (Fig. 2E, F). To detect the inflammatory infiltration in EAE mice, HE staining was performed and showed that there was no difference in the density of infiltrating cells in the control group (Fig. 2G, H). However, compared with that in *SARM1*^*f/f*^ EAE mice, the density of infiltrating cells was significantly decreased in *SARM1*^*GFAP*^-CKO EAE mice (Fig. 2G, H), indicating that the inflammatory infiltration of *SARM1*^*GFAP*^-CKO EAE mice was alleviated. Similarly, there was no difference in density of CD45^+^ (a marker of common leukocyte antigen) cells in the control group. However, the density of CD45^+^ cells in *SARM1*^*GFAP*^-CKO EAE mice was significantly lower than that in *SARM1*^*f/f*^ EAE mice (Fig. 2I, J). Taken together, the above results suggested that conditional knockout of astrocytic *SARM1* delayed onset time, and decreased the loss of neurons and inflammatory infiltration in EAE mice.

### The density of astrocytes and microglia, and proliferation of astrocytes were reduced in the spinal cords of *SARM1*^*GFAP*^-CKO EAE mice

**Fig. 3:**
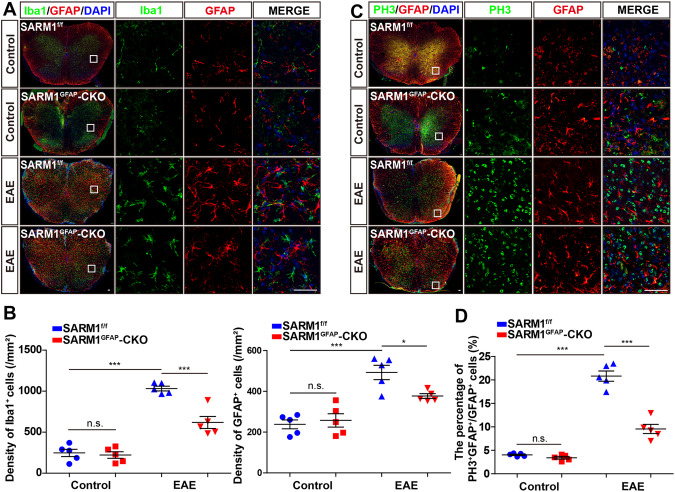
The density of microglia and astrocytes, and proliferation of astrocytes were reduced in the spinal cords of *SARM1*^*GFAP*^-CKO EAE mice. **A** Double immunostaining of Iba1 (green) and GFAP (red) in lumbar spinal cords of *SARM1*^*f/f*^ mice and *SARM1*^*GFAP*^-CKO mice, and *SARM1*^*f/f*^ EAE mice and *SARM1*^*GFAP*^-CKO EAE mice. **B** Quantitative analysis of the density of Iba1^+^ and GFAP^+^ was quantified as shown in **A** (*n* = 5). **C** Double immunostaining analysis of PH3 (green) and GFAP (red) in lumbar spinal cords of *SARM1*^*f/f*^ mice and *SARM1*^*GFAP*^-CKO mice, and *SARM1*^*f/f*^ EAE mice and *SARM1*^*GFAP*^-CKO EAE mice. **D** The quantitative percentage of PH3^+^GFAP^+^ cells in the total number of GFAP^+^ cells as shown in **C** (*n* = 5). Scale bar, 50 μm. The data were mean ± SEM. Student’s t-test, n.s., not significant (*p* > 0.05), *p < 0.05, ***p < 0.001.

Astrocytes and microglia are the key components of MS immunopathology and play an indispensable role in the formation of harmful positive feedback inflammation cycles [32, 33]. Therefore, we tested whether the ablation of astrocytic *SARM1* affected the response of these inflammatory cells. Double immunostaining showed there was no obvious difference in the density of Iba1^+^ microglia and GFAP^+^ astrocytes in the control group (Fig. 3A, B). In the EAE group, the density of Iba1^+^ and GFAP ^+^ cells were obviously increased, and their morphology was more hypertrophic than that in the control group. However, compared with that in *SARM1*^*f/f*^ EAE mice, the density of Iba1^+^ microglia and GFAP^+^ astrocytes were significantly decreased in the lumbar spinal cords of *SARM1*^*GFAP*^-CKO EAE mice (Fig. 3A, B). Previous studies have shown that Aldh1L1 is a better immunohistochemical astrocyte marker than GFAP because it better labels the cell bodies of astrocytes in both white and grey matter [34]. To further determine the changes of astrocytes, we performed double immunostaining of Aldh1L1 and GFAP and found that astrocytes were activated in control EAE mice, but decreased in *SARM1*^GFAP^-CKO EAE mice (Fig. S2A, B). These results suggested that microglia and astrocytes were activated in EAE mice, while astrocytic *SARM1* deletion resulted in less activation of microglia and astrocytes. Furthermore, immunostaining of PH3/Ki67 and GFAP showed that the percentage of PH3^+^GFAP^+^ /GFAP^+^ astrocytes (Fig. 3C, D) and the percentage of Ki67^+^GFAP^+^ /GFAP^+^ astrocytes (Fig. S2C, D) were significantly decreased in *SARM1*^*GFAP*^*-CKO* EAE mice, compared with that in *SARM11*^*f/f*^ EAE mice, which suggested that decrease of astrocyte density might be due to decrease of astrocyte proliferation. Since SARM1 was not expressed in microglia [20], the effect of SARM1 on microglia might be indirect. These results suggested that conditional deletion of astrocytic *SARM1* alleviated the inflammatory response of EAE and reduced the proliferation of astrocytes.

### The demyelination and axonal injury were alleviated in the spinal cords of *SARM1*^*GFAP*^-CKO EAE mice

**Fig. 4:**
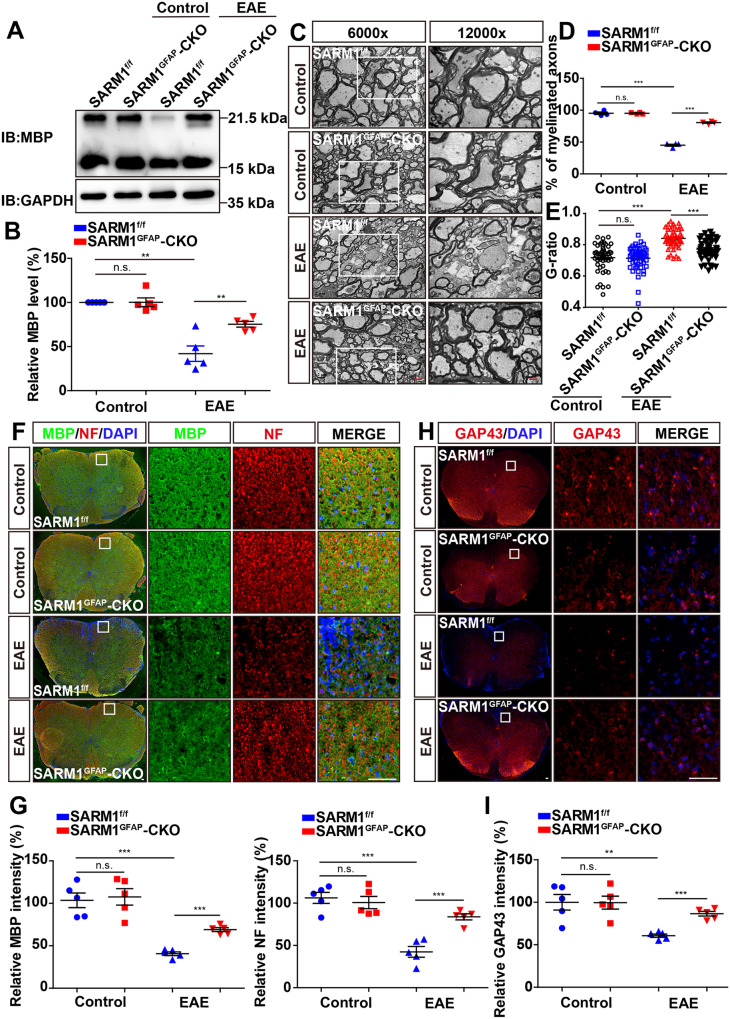
Demyelination and axonal injury were alleviated in the spinal cords of *SARM1*^*GFAP*^-CKO EAE mice. **A** Western blot analysis of MBP expression in spinal cords of *SARM1*^*f/f*^ mice and *SARM1*^*GFAP*^-CKO mice, and *SARM1*^*f/f*^ EAE mice and *SARM1*^*GFAP*^-CKO EAE mice. The band of higher molecular weight may be 21.5 kDa isoform, while that of lower molecular weight may represent 18.5 kDa and 17.2 kDa isoform. **B** Quantitative analysis of MBP was shown in (**A**) (normalized to *SARM1*^*f/f*^ mice, *n* = 5). **C** The typical electron microscopic images of lumbar spinal cords in *SARM1*^*f/f*^ mice and *SARM1*^*GFAP*^-CKO mice, and *SARM1*^*f/f*^ EAE mice and *SARM1*^*GFAP*^-CKO EAE mice. Scale bars, 2 μm (low power image) and 1 μm (high power image). **D** Quantitative analysis of percentage of myelinated axons as shown in **C** (*n* = 4). **E** Quantitative analysis of G-ratio as shown in **C** (*n* = 50, two-way ANOVA with Bonferroni’s post-tests). **F** Double immunostaining MBP (green) and NF (red) in spinal cords of *SARM1*^*f/f*^ mice and *SARM1*^*GFAP*^-CKO mice, and *SARM1*^*f/f*^ EAE mice and *SARM1*^*GFAP*^-CKO EAE mice. **G** Quantitative analysis of the relative expression of MBP and NF as shown in (**F**) (*n* = 5). **H** Immunostaining of GAP43 (red) in spinal cords of *SARM1*^*f/f*^ mice and *SARM1*^*GFAP*^-CKO mice, and *SARM1*^*f/f*^ EAE mice and *SARM1*^*GFAP*^-CKO EAE mice. **I** Quantitative analysis of the relative GAP43 expression as shown in (**H**) (*n* = 5). Scale bar, 50 μm. Data were mean ± SEM. Student’s t-test unless otherwise indicated, n.s., not significant (*p* > 0.05), **p < 0.01, ***p < 0.001.

In addition to inflammation and neuronal death, demyelination and axonal injury are also pathological features of MS/EAE [4]. We next tested whether *SARM1* ablation in astrocytes affected the demyelination and axonal injury in EAE model. Myelin basic protein (MBP) is a common marker of myelin protein. It has been reported that the 21.5 kDa isoform of MBP occurs in the early stage of myelin formation or in areas of remyelination of injured white matter, while the 18.5 kDa isoform was rich in the mature myelin [35]. As shown in Fig. 4A, B, 21.5 kDa isoform of MBP was more significantly affected in *SARM1*^*GFAP*^-CKO mice, while the 18.5 kDa and 17.2 kDa isoforms were less affected, which revealed that *SARM1* deletion might had more effect on myelin formation and regeneration, and less effect on mature myelin in EAE mice. Moreover, there was no difference in the level of MBP protein in *SARM1*^*f/f*^ mice and *SARM1*^*GFAP*^-CKO mice. However, the protein level of 21.5 kDa isoform of MBP was significantly decreased in *SARM1*^*f/f*^ EAE mice, compared with that in *SARM1*^*f/f*^ mice, whereas the protein level of 21.5 kDa isoform of MBP of *SARM1*^*GFAP*^-CKO EAE mice was significantly higher than that in *SARM1*^*f/f*^ EAE mice, which indicated myelin formation and regeneration instead of mature myelin might be the target of SARM1 in EAE mice. Furthermore, electron microscopy showed that the demyelinating phenotype in *SARM1*^*GFAP*^-CKO EAE mice was significantly alleviated, compared with that in *SARM1*^*f/f*^ EAE mice (Fig. 4C–E). Besides, double immunostaining showed that the intensity of MBP and NF (neurofilament heavy polypeptide) in *SARM1*^*f/f*^ EAE mice was obviously decreased, compared with that in *SARM1*^*f/f*^ mice. Similarly, the intensity of MBP and NF in *SARM1*^*GFAP*^ -CKO EAE mice was significantly higher than that in *SARM1*^*f/f*^ EAE mice (Fig. 4F, G). Finally, immunostaining of GAP43 (another axonal marker) showed that the intensity of GAP43 in *SARM1*^*GFAP*^-CKO EAE mice was significantly higher than that in *SARM1*^*f/f*^ EAE mice (Fig. 4H, I). In conclusion, these results suggested that the deletion of astrocytic *SARM1* alleviated the demyelination and axonal injury in EAE mice.

### EAE was relieved with later onset, fewer neuronal death, demyelination, axonal injury and less inflammatory infiltration in *SARM1*^*Aldh1L1*^-CKO mice

**Fig. 5:**
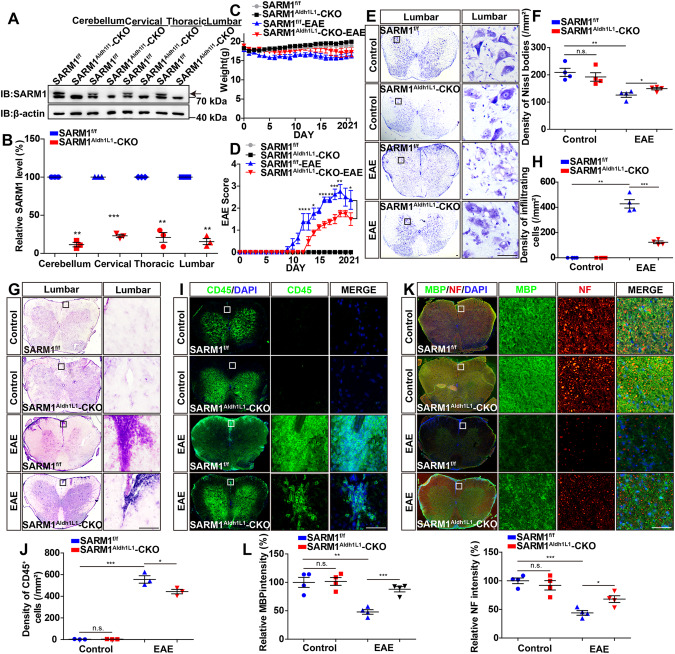
EAE was relieved with later onset, fewer neuronal death, demyelination, axonal injury and less inflammatory infiltration in *SARM1*^*Aldh1L1*^-CKO mice. **A** Western blot detected the expression of SARM1 in the cerebellum, cervical, thoracic and lumbar spinal cords of *SARM1*^*f/f*^ mice and *SARM1*^*Aldh1L1*^-CKO mice. **B** Quantification of protein levels of SARM1 as shown in **A** (normalized to *SARM1*^*f/f*^ mice, *n* = 3). **C** The weight of *SARM1*^*f/f*^ EAE and *SARM1*^*Aldh1L1*^-CKO EAE mice ranged from 0 to 21 dpi (*n* = 4 mice, two-way ANOVA with Bonferroni’s post-tests). **D** The EAE score of *SARM1*^*f/f*^ and *SARM1*^*Aldh1L1*^-CKO mice ranged from 0 to 21 dpi (*n* = 4 mice, two-way ANOVA with Bonferroni’s post-tests). **E** Typical images of Nissl staining in lumbar spinal cords of *SARM1*^*f/f*^ and *SARM1*^*Aldh1L1*^-CKO mice, and *SARM1*^*f/f*^ EAE and *SARM1*^*Aldh1L1*^-CKO EAE mice. **F** Quantitative analysis of the density of Nissl bodies as shown in **E** (*n* = 4). **G** The typical images of HE staining of lumbar spinal cords in *SARM1*^*f/f*^ and *SARM1*^*Aldh1L1*^-CKO mice, and *SARM1*^*f/f*^ EAE and *SARM1*^*Aldh1L1*^-CKO EAE mice. **H** Quantitative analysis of the density of infiltrating cells as shown in **G** (*n* = 4). **I** The typical images of CD45^+^ immunostaining in lumbar spinal cords of *SARM1*^*f/f*^ and *SARM1*^*Aldh1L1*^-CKO mice, and *SARM1*^*f/f*^ EAE and *SARM1*^*Aldh1L1*^-CKO EAE mice. **J** Quantitative analysis of the density of CD45^+^ cells as shown in **I** (*n* = 3). **K** The typical images of immunostaining of MBP (green) and NF (red) in lumbar spinal cords of S*ARM1*^*f/f*^ and *SARM1*^*Aldh1L1*^-CKO mice, and *SARM1*^*f/f*^ EAE and *SARM1*^*Aldh1L1*^-CKO EAE mice. **L** Quantitative analysis of the intensity of MBP and NF as shown in **K** (*n* = 4). Scale bar, 50 μm. The data were mean ± SEM. Student’s t-test unless otherwise indicated, n.s., not significant (*p* > 0.05), *p < 0.05, **p < 0.01, ***p < 0.001.

Because aldehyde dehydrogenase family member 1L1 (Aldh1L1), specifically expressed by astrocytes, but not by oligodendrocytes or neurons [34], is a recognized cell type-specific immunohistochemical marker for astrocytes [36], *Aldh1L1-Cre* is used as a more specific marker for astrocytes than GFAP in the forebrain and spinal cords [37]. Therefore, *SARM1*^*Aldh1L1*^-CKO mice were used to further exclude the interference of SARM1 in neurons and other glial cells and to confirm the function of SARM1 in astrocytes. Western blot showed that SARM1 was efficiently knockout in the cerebellum, cervical, thoracic, and lumbar spinal cords of *SARM1*^*Aldh1L1*^-CKO mice (Fig. 5A, B), which indicated that our *SARM1*^*Aldh1L1*^-CKO mice were successfully constructed.

In EAE model, we found that the body weight of the control mice and EAE mice has no difference (Fig. 5C) and the clinical score of EAE was significantly lower in *SARM1*^*Aldh1L1*^-CKO EAE mice than that in *SARM1*^*f/f*^ EAE mice (Fig. 5D). Next, Nissl staining revealed that there was no difference in the density of Nissl bodies between *SARM1*^*f/f*^ mice and *SARM1*^*Aldh1L1*^-CKO mice. However, the loss of Nissl bodies in *SARM1*^*Aldh1L1*^-CKO EAE mice was significantly decreased, compared with that in *SARM1*^*f/f*^ EAE mice (Fig. 5E, F). Furthermore, compared with that in *SARM1*^*f/f*^ EAE mice, the density of infiltrating cells (Fig. 5G, H) and CD45^+^ cells (Fig. 5I, J) was significantly decreased in *SARM1*^*Aldh1L1*^-CKO EAE mice, indicating that ablation of astrocytic *SARM1* could alleviate inflammatory infiltration in EAE. Furthermore, the intensity of MBP and NF in *SARM1*^*Aldh1L1*^-CKO EAE mice was also significantly higher than that in SARM1^*f/f*^ EAE mice (Fig. 5K, L). Taken together, the above results further suggested that deletion of astrocytic *SARM1* delayed onset time, relieved demyelination, and decreased neuronal death and inflammatory infiltration in EAE mice.

### GDNF was upregulated in astrocytes, and inhibited NF-κB signaling in *SARM1*^*GFAP*^-CKO EAE mice

**Fig. 6:**
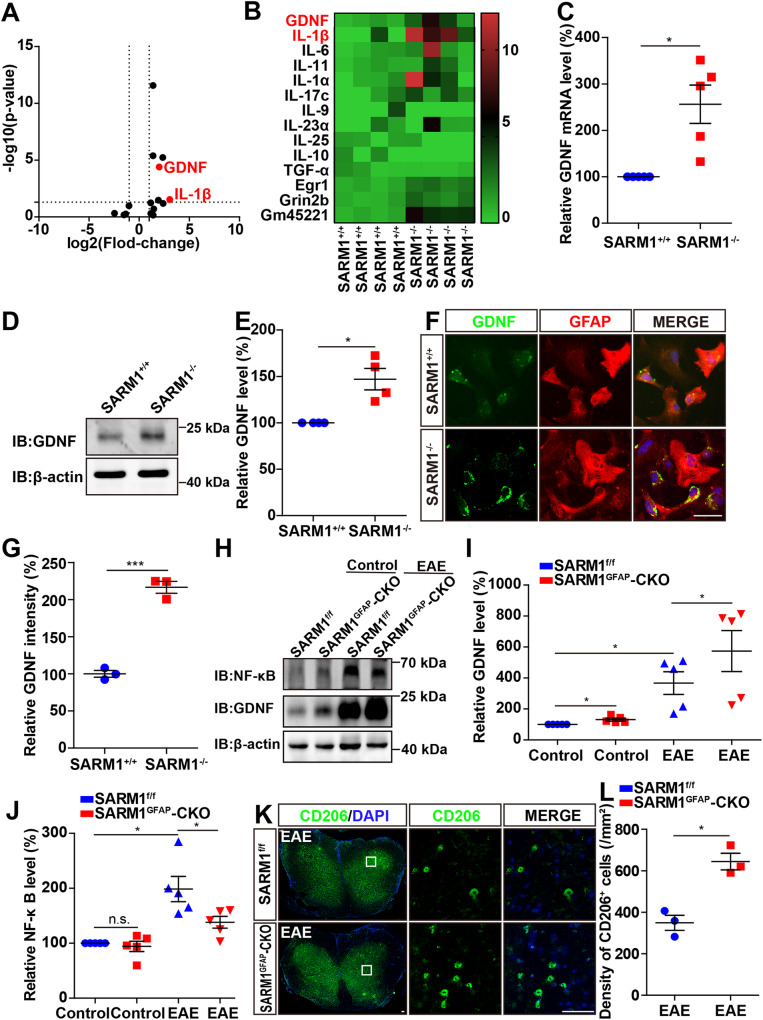
GDNF was upregulated in astrocytes and inhibited NF-κB signaling in *SARM1*^*GFAP*^*-CKO* EAE mice. **A**, **B** The heatmap and volcano plot of differential mRNAs in *SARM1*^*+/+*^ and *SARM1*^*-/-*^ astrocytes (*n* = 4). **C** Analysis of qPCR showed the relative mRNA level of GDNF in *SARM1*^*+/+*^ and *SARM1*^*-/-*^ astrocytes (*n* = 5). **D** Western blot analysis of expression of GDNF in *SARM1*^*+/+*^ and *SARM1*^*-/-*^ astrocytes. **E** Quantitative analysis of relative expression of GDNF was shown in **D** (normalized to *SARM1*^*+/+*^, *n* = 4). **F** The typical images of immunostaining of GDNF (green) and GFAP (red) of *SARM1*^*+/+*^ and *SARM1*^*-/-*^ astrocytes. Scale bar, 50 μm**. G** Quantitative analysis of intensity of GDNF as shown in **F** (*n* = 3). **H** Western blot analysis of the expression of GDNF and NF-κB in *SARM1*^*f/f*^ mice and *SARM1*^*GFAP*^-CKO mice, and *SARM1*^*f/f*^ EAE mice and *SARM1*^*GFAP*^-CKO EAE mice. **I** Quantitative analysis of relative GDNF as shown in **H** (normalized to *SARM1*^*f/f*^ mice, *n* = 5). **J** Quantitative analysis of NF-κB as shown in **H** (normalized to *SARM1*^*f/f*^ mice, *n* = 5). **K** Typical images of immunostaining of CD206^+^ cells of lumbar spinal cords in *SARM1*^*f/f*^ EAE mice and *SARM1*^*GFAP*^-CKO EAE mice. **L** Quantitative analysis of the density of CD206^+^ cells as shown in **K** (normalized to *SARM1*^*f/f*^ EAE mice, *n* = 3). Scale bar, 50 μm. The data were mean ± SEM. Student’s t-test, n.s., not significant (*p* > 0.05), *p < 0.05, ***p < 0.001.

How can ablation of astrocytic *SARM1* lead to the relief of EAE phenotypes? RNA-sequencing showed that GDNF in primary cultured astrocytes from *SARM1*^*GFAP*^-CKO mice was higher than that from *SARM1*^*f/f*^ mice (Fig. 6A, B). GDNF is a member of the TGF-β superfamily [38], first discovered in rat glial cells, which can mediate the growth, differentiation and migration of neurons and is necessary for the formation of axons and dendrites [39]. Further quantitative real-time PCR (q-PCR), western blot and immunocytochemistry showed that GDNF was indeed upregulated in *SARM1*^*-/-*^ astrocytes (Fig. 6C–G).

In addition, it is reported that GDNF promotes the transformation of M1 microglia to M2 microglia by inhibiting NF-κB and promoting the PI3K/AKT signaling pathway [40]. To observe whether upregulation of GDNF in *SARM1*^*GFAP*^-CKO EAE mice regulates the above-mentioned pathways, we examined the changes of GDNF and NF-κB in the protein level. Interestingly, our results showed that the expression of GDNF was upregulated in *SARM1*^*GFAP*^*-*CKO mice, compared with that in *SARM1*^*f/f*^ mice and the expression of GDNF in *SARM1*^*GFAP*^-CKO EAE mice was significantly higher than that in *SARM1*^*f/f*^ EAE mice. In addition, the expression of GDNF was low at the basal level, but it was significantly increased in EAE mice (Fig. 6H, I), which might be related to the increased secretion of GDNF to promote the survival of neurons under disease conditions [40]. Meanwhile, the protein level of NF-κB in EAE mice was upregulated, while it was significantly lower in *SARM1*^*GFAP*^-CKO EAE mice than that in *SARM1*^*f/f*^ EAE mice (Fig. 6H, J). Next, CD206 (M2 microglial marker) immunostaining showed that the density of CD206^+^ cells was increased in the *SARM1*^*GFAP*^-CKO EAE mice, compared with that in *SARM1*^*f/f*^ EAE mice (Fig. 6K–L). Taken together, these results suggested that GDNF was upregulated in astrocytes, inhibited NF-κB signaling, and promoted the polarization of M2 microglia in *SARM1*^*GFAP*^-CKO EAE mice, which may be responsible for the neuroprotective effect of astrocytic *SARM1* deletion in EAE mice.

### Deletion of astrocytic *SARM1* performed the neuroprotective effects through GDNF signaling in EAE mice

**Fig. 7:**
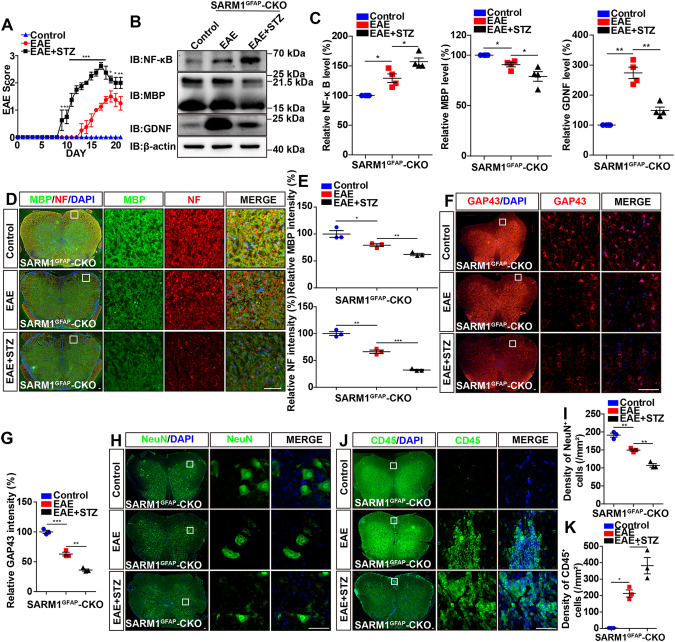
Inhibition of GDNF by STZ worsened the EAE deficits in *SARM1*^*GFAP*^-CKO EAE mice. **A** The EAE score of *SARM1*^*GFAP*^-CKO mice, *SARM1*^*GFAP*^-CKO EAE mice and STZ-treated *SARM1*^*GFAP*^-CKO EAE mice during EAE modeling process (*n* = 4). **B** Western blot detected NF-κB, GDNF and MBP in the spinal cords of *SARM1*^*GFAP*^-CKO mice, *SARM1*^*GFAP*^-CKO EAE mice and STZ-treated *SARM1*^*GFAP*^-CKO EAE mice. **C** Quantification of protein levels of NF-κB, GDNF and MBP as shown in **B** (*n* = 4, normalized to β-actin). **D** Immunostaining of MBP (green) and NF (red) in spinal cords of *SARM1*^*GFAP*^-CKO mice, and *SARM1*^*GFAP*^-CKO EAE mice and STZ-treated *SARM1*^*GFAP*^-CKO EAE mice. **E** Quantification of the relative intensity of MBP and NF (normalized to *SARM1*^*GFAP*^-CKO mice, *n* = 3). **F** Immunostaining of GAP43 (red) in spinal cords of *SARM1*^*GFAP*^-CKO mice, and *SARM1*^*GFAP*^-CKO EAE mice and STZ-treated *SARM1*^*GFAP*^-CKO EAE mice. **G** Quantification of the relative intensity of GAP43 as shown in **F** (normalized to *SARM1*^*GFAP*^-CKO mice, *n* = 3). **H** Typical images of immunostaining of NeuN^+^ cells in spinal cords of *SARM1*^*GFAP*^*-CKO* mice, and *SARM1*^*GFAP*^-CKO EAE mice and STZ-treated *SARM1*^*GFAP*^-CKO EAE mice. **I** Quantitative analysis of the density of NeuN^+^ cells as shown in **H** (*n* = 3). **J** Typical images of immunostaining of CD45^+^ cells in spinal cords of *SARM1*^*GFAP*^-CKO mice, and *SARM1*^*GFAP*^-CKO EAE mice and STZ-treated *SARM1*^*GFAP*^-CKO EAE mice. **K** Quantitative analysis of the density of CD45^+^ cells as shown in **J** (*n* = 3). Scale bar, 50 μm. Data were mean ± SEM., One-way ANOVA with Bonferroni’s post-tests, *p < 0.05, **p < 0.01, ***p < 0.001.

To examine whether *SARM1* deletion in astrocytes performed neuroprotective effects through GDNF signaling, we next checked whether inhibition of GDNF worsened EAE phenotypes in *SARM1*^*GFAP*^-CKO EAE mice. Previous studies have suggested that STZ reduces the expression of GDNF in astrocytes [30]. As expected, primary astrocytes from *SARM1*^*GFAP*^-CKO mice were cultured and treated with STZ (100 μM) for 24 h, and the expression of GDNF in astrocytes was indeed reduced by STZ treatment (Supplementary Fig. S3A, B). Interestingly, STZ significantly worsened the clinical scores of *SARM1*^*GFAP*^-CKO EAE mice (Fig. 7A). We found that GDNF was significantly decreased after injected with STZ, while the expression of MBP protein was decreased and the expression of NF-κB protein was increased in *SARM1*^*GFAP*^-CKO EAE mice (Fig. 7B, C). Moreover, the intensity of GAP43, MBP and NF was significantly decreased in *SARM1*^*GFAP*^-CKO EAE mice after injected with STZ (Fig. 7D–G). Meanwhile, we found that the density of NeuN^+^ cells was decreased and the density of CD45^+^ inflammatory cells was significantly increased in STZ-treated *SARM1*^*GFAP*^-CKO EAE mice, compared with control-treated *SARM1*^*GFAP*^-CKO EAE mice (Fig. 7H–K), suggesting that deletion of astrocytic *SARM1* performed the neuroprotective effects through GDNF signaling in EAE mice.

## Discussion

**Fig. 8:**
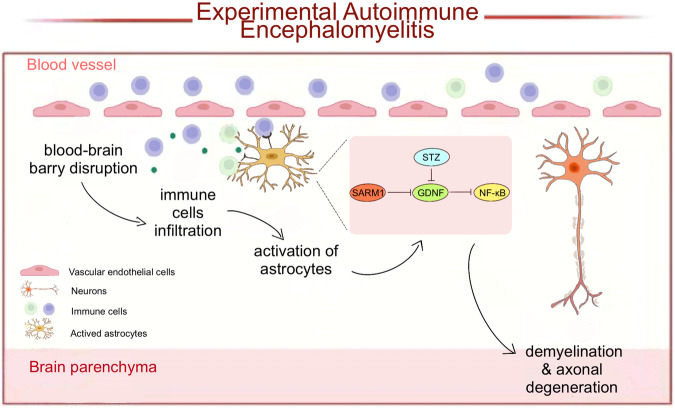
A working model of astrocytic SARM1’s function in EAE mice. SARM1 is upregulated in the astrocytes of spinal cords in EAE. Astrocytic SARM1 inhibits the expression of GDNF, which promotes the neuronal death, neuroinflammation and demyelination in EAE through NF-κB signaling.

In this study, we provided evidence for the role of SARM1 in EAE and proposed a working model (Fig. 8). In this model, SARM1 is upregulated in astrocytes of EAE mice, and somehow inhibits the expression of GDNF, which may contribute to neuroinflammation and demyelination by activating NF-κB signaling. Interestingly, in EAE model, conditional knockout of astrocytic *SARM1* indeed inhibits neuronal death, neuroinflammation, and improves the behavioral recovery of motor function.

SARM1 is usually present in axons in an inactive state under normal conditions, but can be activated when the body is injured [28], which is consistent with our results (Fig. 1A, B). Regarding how SARM1 regulates the phenotype of EAE, mRNA sequencing revealed that GDNF was increased in *SARM1*^*-/-*^ astrocytes (Fig. 6A, B). Carty’s research team has shown the removal of *SARM1* can inhibit the expression of IL-1β depending on the activation of the NLRP3 inflammasome [41]. Previous studies have found that the NLRP3 inflammasome was indeed activated in EAE model [42]. In addition, in rat neuron-glia co-culture, damaged dopaminergic neurons induce astrocytes to release GDNF through IL-1β [43], which indicates that the expression of GDNF in astrocytes is stimulated by inflammation and can provide neurotrophic support for injured neurons under the conditions of CNS inflammation [44, 45]. Another example is that IL-1β stimulates GDNF expression in C6 glioma cells through the inhibitor kappa B (IκB), p38 MAP kinase, p44/p42 MAP kinase and JAK-STAT3 pathways [46]. In our RNA sequencing results, we surprisingly found that IL-1β was higher in *SARM1*^*-/-*^ astrocytes than that in *SARM1*^*+/+*^ astrocytes (Fig. 6A, B). Therefore, we suspect that the knockout of *SARM1* in astrocytes may increase GDNF by increasing the expression of IL-1β. In the future, further studies need to be performed to test this possibility.

The release of GDNF is expected to have therapeutic potential for neurodegenerative diseases, traumatic and inflammatory brain injuries [46]. In our study, inflammation was reduced in *SARM1*^*GFAP*^-CKO EAE mice and *SARM1*^*Aldh1L1*^-CKO EAE mice, which may be related to the increase of GDNF that can inhibit the NF-κB inflammation signaling pathway (Fig. 6H–J) and increase the density of M2 microglia (Fig. 6K, L). It is reported that AKT plays an important role in the nervous system and inhibits the SARM1-mediated axonal injury pathway [47]. We speculate that changes of demyelination and axon damage may be related to AKT. Although there was no direct evidence in our experiments that increase of the intensity of MBP and NF in *SARM1*^*GFAP*^-CKO EAE mice and *SARM1*^*Aldh1L1*^-CKO EAE mice is indeed the reason for improving the recovery of motor function, a large amount of evidence indicates that increase of the intensity MBP and NF is associated with the recovery of motor function [48]. Therefore, we speculate that increased intensity of MBP and NF in *SARM1*^*GFAP*^-CKO EAE mice and *SARM1*^*Aldh1L1*^-CKO EAE mice, compared with that in *SARM1*^*f/f*^ EAE mice may be the reason for the improvement in motor function recovery. Finally, after inhibition of the expression of GDNF by intraperitoneal injection of STZ, the improved symptoms of *SARM1*^*GFAP*^-CKO EAE mice were suppressed, which further proved that SARM1 regulated EAE through GDNF signaling. How astrocytic SARM1 regulates the expression of GDNF in EAE needs to be further studied in the future.

In summary, our findings reveal an unrecognized function of removal of astrocytic SARM1 to prevent axonal injury, demyelination and neuroinflammation, and reveal its possible regulatory pathways, which will help to develop new therapies for MS.

## Materials and methods

### Animals

The *SARM1* allele (*SARM1*^*f/f*^) mice were generated as previously described [48]. *GFAP-Cre* mice (from Jackson Laboratory) were crossed with the floxed *SARM1*^*f/f*^ mice to get *SARM1*^*GFAP*^-CKO mice. *SARM1*^*Aldh1L1*^-CKO mice were generated by crossing the floxed *SARM1*^*f/f*^ mice with *Aldh1L1-Cre* transgenic mice (gifted from Zilong Qiu’s lab, Institute of Neuroscience, Chinese Academy of Sciences). These mice were based on the C57BL/6 background and genotyped by PCR. All mice were housed in a specific pathogen-free (SPF) facility at Wenzhou Medical University under controlled temperature (22–25 °C) with a 12-h light-dark cycle, with water and food provided ad libitum. All animal experiments strictly follow the guidelines of the Laboratory Animals Ethics Committee of Hangzhou Normal University and Wenzhou Medical University.

### Establishment and treatment of EAE model in mice

The choice of sample size was based on experience with previously published EAE models and studies. The EAE model is as described previously [49]. In short, female mice weighing 18 g to 20 g were injected with 0.2 mg of emulsified MOG_35–55_ (1:1) (HY-P1240A, MedChemExpress) in Freund’s complete adjuvant containing 8 mg/ml Mycobacterium tuberculosis (strain H37RA; Difco, USA). Two intraperitoneal injections of 300 ng of pertussis toxin (516561, Sigma) were respectively given at 0 and 48 h after immunization. The mice of the control group were injected with PBS in the same manner. Then the score (0.5 = tail weakness, 1 = tail paralysis, 2 = faltering gait with hind limb weakness, 2.5 = unilateral hind limb paralysis, 3 = bilateral hind limb paralysis, 4 = hind limb paralysis with forelimb weakness, 5 = near death) and body weight were recorded from days 0 to 21.

STZ (FS0266, FuShen) was dissolved in sodium citrate solution (C1013, Solarbio) and intraperitoneally injected with 150 mg/kg to mice starved for more than 12 h. Every mouse was randomly assigned to the experimental groups. Evaluation of genotype and experimental conditions was blinded.

### Western blotting

Mice were anesthetized by intraperitoneal injection of tribromoethanol. The lumbar spinal cords were obtained after cardiac perfusion with PBS and then ground in a lysis solution containing RIPA buffer (p0013b, beyotime), 100 mM NaF, 100 mM Na3VO4 and 100 mM PMSF three times for 45 s and left at 4 °C for 30 min. After centrifugation for 20 min, the supernatant was mixed with 5x loading buffer and then heated at 100 °C for 10 min to obtain protein samples. The protein samples were separated by 8%, 10% and 12% twelve alkyls sulfate-polyacrylamide gel electrophoresis and transferred to the PVDF membrane (Pierce Chemical Company, Illinois, USA). The PVDF membrane was sealed in 1x protein-free rapid blocking buffer for 0.5 h at room temperature, and then mixed with different primary antibodies at 4 °C. The primary antibodies included mouse anti-MBP (1:1000, ab62631, abcam), mouse anti-β-actin (1:10000, A5316, Sigma-Aldrich), mouse anti-GAPDH (1:5000, #T0004, Affinity), rabbit anti-NF-κB (p65) (1:1000, ab16502, abcam), mouse anti-GDNF (1:500, sc-13147, SANTA CRUZ), rabbit anti-SARM1 (1:1000, GTX131411, GeneTex). On the second day, after 30 min of TBST washing, the PVDF membrane was incubated with horseradish peroxidase-conjugated secondary antibody (1:5000) at room temperature for 1.5 h. The secondary antibodies included goat anti-mouse IgG-HRP (#31460, Pierce, 1:5000) and goat anti-rabbit IgG-HRP (#31420, Pierce, 1:5000). The positive signal was generated by ECL detection kit (1705061, Bio-Rad, USA), and the image information was analyzed using the Quantity One software (Bio-Rad, USA).

### Hematoxylin-Eosin (HE) staining

In brief, the mice were perfused with 0.1 M PBS and 4% PFA after anesthetization. Then the spinal cords of the mice were immersed in 4% PFA for 24 h and transferred to 15% sucrose solution until they sank. In the same way, the spinal cords were transferred to 30% sucrose solution and dehydrated again. Subsequently, the spinal cords were embedded with OCT and coagulated at -20 °C. The spinal cords were cut into 20 μm-thick sections using a freezing microtome (Thermo, USA) and then pasted on the adhesive glass slides. After being stained with hematoxylin for 1 min, the slides were washed in double distilled water, cultured in acidic liquid alcohol for 30 s, stained with eosin for 50 s, dehydrated with 95% ethanol and 100% ethanol for 1 min, and finally soaked in xylene for 10 min, fixed with neutral resin. Images were collected by microscope (Nikon, Tokyo, Japan) at room temperature and quantitatively analyzed by Image J software (Media Cybernetics, Bethesda, MD, USA).

### Nissl’s staining

Sections of the spinal cords were obtained as previously described. The 20 μm thick sections were soaked in 0.1% cresol violet at room temperature for 6 min, then washed with double distilled water for 5 min, dehydrated with 95% ethanol and 100% ethanol, respectively, and finally soaked in xylene for 10 min and fixed with neutral resin. The images were collected by microscope (Nikon, Tokyo, Japan) at room temperature and analyzed quantitatively by Image J.

### Immunocytochemistry

After being washed three times with PBS, cultured cells were fixed in 4% paraformaldehyde (PFA) for 15 min, and then soaked in a PBS solution containing 0.1 Triton X-100, 1% fetal bovine serum (FBS, GIBCO), and 5% BSA for 1.5 h. Subsequently, the cells were incubated with multiple primary antibodies overnight at 4 °C, and washed three times with PBS on the second day, and then incubated with appropriate secondary antibodies (1:1000, Invitrogen) and DAPI (1:1000, Sigma-Aldrich) in 5% BSA for 1.5 h at room temperature. The primary antibodies include rabbit anti-GFAP (1;500, bs-0199R, Bioss), mouse anti-GDNF (1:500, sc-13147, SANTA CRUZ). The secondary antibodies included donkey antimouse Alexa Fluor488 (A21202, Invitrogen,1:1000), donkey antirabbit Alexa Fluor546 (A10040, Invitrogen, 1:1000). Finally, images were captured with a microscope (Nikon, Tokyo, Japan) at room temperature and analyzed by Photoshop (Adobe) and Image J.

### Immunofluorescence

After being washed three times with PBS, the slices of the spinal cords were fixed in 4% PFA for 30 min and then incubated with 5% BSA plus 0.3% Triton X-100 for 1.5 h. Next, the slides of spinal cords were incubated with multiple primary antibodies overnight at 4 °C, and washed three times with PBS on the second day, and then incubated with appropriate secondary antibodies and DAPI in 5% BSA for 1.5 h at room temperature. The primary antibodies include mouse anti-GFAP (1:500, MAB360, Millipore), rabbit anti-GFAP (1;500, bs-0199R, Bioss), mouse anti-NeuN (1:500, ab104224, abcam), rabbit anti-CD45 (1:500, ab10558, abcam), mouse anti-MBP (1:500, ab62631, abcam), rabbit anti-NF (1:500, ab8135, abcam), mouse anti-PH3 (1:500, ab14955, abcam), rabbit anti-GAP43 (1:500, ab16053, abcam), rabbit anti-SARM1 (1:500, ab226930, abcam), goat anti-Iba1 (1:500, ab5076, abcam), rabbit anti-Iba1 (1;200, ab153696, abcam), rabbit anti-Aldh1L1 (1:200, ab177483, abcam), rabbit anti-Ki67 (1:200, #9129, Cell Signaling Technology), rabbit anti-Mannose Receptor (CD206, 1:200, ab64693, abcam). Secondary antibodies included donkey anti-rabbit Alexa Fluor488 (A21206, Invitrogen, 1:1000), donkey anti-mouse Alexa Fluor488, donkey anti-rabbit Alexa Fluor546, donkey anti-mouse Alexa Fluor546 (A10036, Invitrogen, 1:1000), donkey anti-goat Alexa Fluor488 (A11055, Invitrogen, 1:1000). Finally, images were captured with a microscope (Nikon, Tokyo, Japan) at room temperature and analyzed by Photoshop (Adobe) and Image J.

### Culture of astrocytes

In short, the cerebral cortex and hippocampus of newborn mice (P1-P3) were dissected and chopped in PBS under a microscope. After digestion with 0.25% trypsin (GIBCO) at 37 °C for 15 min, the tissue was stopped digesting by adding DMEM medium containing 10% FBS. Then the tissue was mechanically destroyed to obtain single cell suspension. The cells were then seeded in a poly lysine (0.1 mg/ml, sigma Aldrich) coated culture flask. After the cells were cultured in a 37°, 5% CO_2_ incubator for 6–10 d, microglia and oligodendrocytes were removed by shaking at 250 rpm for 4–6 h. Astrocytes were then isolated and plated into poly-D-lysine coated Petri dishes or coverslips. The purity of GFAP^+^ cells was over 94%.

### RNA sequencing and functional enrichment analysis

The total RNA was obtained from cultured *SARM1*^*+/+*^ astrocytes and *SARM1*^*-/-*^ astrocytes by using the RNeasy Mini kit (Qiagen). The conditions for sequencing RNA samples are A260:A280 ratio>1.8 and A260:A230 ratio>2.0. The method and analysis of sequence have been described previously [48]. In short, genes that meet the criteria of fold change >2 and adjusted p-value <0.05 are considered to be genes with disparity significance, which were analyzed using heatmap and Kyoto Encyclopedia of Genes and Genomes (KEGG) ontology enrichment.

### Quantitative reverse transcription polymerase chain reaction (qRT-PCR)

The operation of extracting total RNA from cultured astrocytes was as described above. Reverse transcription of RNA into cDNA is done by SuperScript™ One-Step Reverse Transcription Kit (#10928–034, Invitrogen). The expression level of GDNF mRNA was quantified on a real-time PCR detection system (Applied Biosystems, USA) using iTaq™ Universal SYBR® Green Supermax (172–5122, Bio-Rad). β-actin was used as endogenous control. The analysis method was described previously [49]. The primers were synthesized by Sangon Biotech, and the sequence is as follows: GDNF-F:5’-GAGGCATCTGGTCACAGCGATAAG-3’, GDNF-R:5’-ATGGCAGGCACTTGGAGTCTTAAC-3’[40]; β-actin-F:5’-GTGACGTTGACATCCGTAAAGA-3’, β-actin-R:5’-GCCGGACTCATCGTACTCC-3’ [50].

### Electron Microscopy and Quantitative Analysis

The method was followed as described previously [49]. Briefly, after anesthesia, mice were immediately dissected on ice and spinal cords were fixed with 2.5% glutaraldehyde overnight. Then the samples were immersed in 2 ml PBS (0.1 M, pH 7.0) for 3 times (10 min each time), fixed for 1.5 h with 1% OsO4 (SPI-CHEM) made from PBS, and then immersed in PBS for 3 times (10 min each time). After that, the samples were dehydrated with ethanol (30%, 50%, 70%, 80%, 90%, and 95%) for 15 min, then dehydrated with 95% acetone for 20 min, and then transferred to 100% acetone (Sinopharm Chemical Reagent Co., Ltd.) for 20 min. The samples were then placed in a mixture of 100% acetone and final spur resin 1:1 (SPI-CHEM) at room temperature for 1 h, then transferred to a 1:3 mixture of acetone and final resin for 3 h, and transferred to the final spur resin mixture (SPI-CHEM) overnight. Then the samples were embedded in spur resin (SPI-CHEM), heated at 70 °C for more than 9 h, sliced on LEICA EM UC7 ultra-thin microtome, stained with uranyl acetate and basic lead citrate (Sinopharma Chemical Reagent Co., Ltd.) for 5–10 min, respectively, and observed and photographed under Hitachi H-7650 transmission electron microscope. The quantitative analysis of image is completed by image J. The ratio of the length of the axon diameter to the length of the myelinated fiber is the G-ratio of the myelinated fiber [51].

### Data analysis and statistics

As for the densities calculated of astrocytes and microglia, GFAP^+^ astrocytes and Iba1^+^ microglia of the whole spinal cord cross sections were counted and then divided by the areas of the spinal cord slices analyzed by image J. All data mean the average ± SEM of more than three independent experiments. GraphPad prism5 and image J were used for statistical analysis. Student’s *t*-test and ANOVA with Bonferroni’s post-tests were used. The statistical significance was set as *P* < *0.05*. Other details have been recorded in the figure legends.

### Supplementary information

Reproducibility checklist

Supplementary material (Figure S1 Figure S2 Figure S3)

Graphical Abstract

## Data Availability

The datasets generated and analysed during this study are available from the corresponding author on reasonable request. The raw sequence data have been deposited in the Genome Sequence Archive under accession number CRA007452.
