# Correction: Astrocytic SARM1 promotes neuroinflammation and axonal demyelination in experimental autoimmune encephalomyelitis through inhibiting GDNF signaling

**DOI:** 10.1038/s41419-024-06517-9

**Published:** 2024-03-14

**Authors:** Lingting Jin, Jingjing Zhang, Xin Hua, Xingxing Xu, Jia Li, Jiaojiao Wang, Mianxian Wang, Huitao Liu, Haoyu Qiu, Man Chen, Xu Zhang, Ying Wang, Zhihui Huang

**Affiliations:** 1 Department of Neurology, The First Affiliated Hospital of Wenzhou Medical University, Wenzhou, Zhejiang China; 2 School of Basic Medical Sciences, Wenzhou Medical University, Wenzhou, 325035 Zhejiang China; 3 School of Pharmacy, and Department of Neurosurgery of the Affiliated Hospital,, Hangzhou Normal University, Hangzhou, 311121 Zhejiang China; 4 Clinical Research Center, Affiliated Hangzhou First People’s Hospital, Zhejiang University School of Medicine, Hangzhou, Zhejiang 310003 China

**Keywords:** Astrocyte, Neuroimmunology

Correction to: *Cell Death and Disease* 10.1038/s41419-022-05202-z, published online 02 September 2022

In this article, in Fig. 2C, the Nissl staining of SARMl^f/f^ as a control and Fig. 5E were identical.
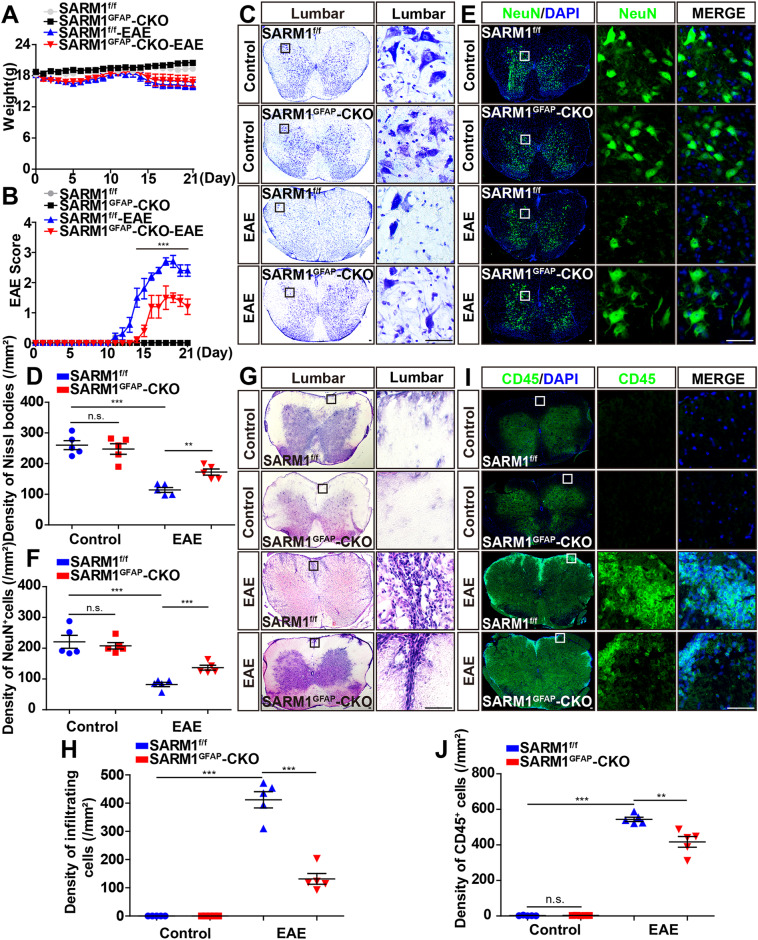


**Fig. 2 EAE was relieved with later onset, less inflammatory infiltration, and fewer neuronal death in** ***SARM1***^***GFAP***^**-CKO mice. A** The weight of *SARM1*^*f/f*^ mice and *SARM1*^*GFAP*^-CKO mice, and *SARM1*^*f/f*^ EAE mice and *SARM1*^*GFAP*^-CKO EAE mice ranged from 0 to 21 dpi (*n* = 5, two-way ANOVA with Bonferroni’s post-tests). **B** The EAE score of *SARM1*^*f/f*^ and *SARM1*^*GFAP*^-CKO mice ranged from 0 to 21 dpi (*n* = 5, two-way ANOVA with Bonferroni’s post-tests). **C** Typical images of Nissl staining in the lumbar spinal cords of *SARM1*^*f/f*^ mice and *SARM1*^*GFAP*^-CKO mice, and *SARM1*^*f/f*^ EAE mice and *SARM1*^*GFAP*^-CKO EAE mice. **D** Quantitative analysis of density of Nissl bodies as shown in (**C**) (*n* = 5). **E** Typical images of NeuN^+^ immunostaining in the lumbar spinal cords of *SARM1*^*f/f*^ and *SARM1*^*GFAP*^-CKO mice, and *SARM1*^*f/f*^ EAE and *SARM1*^*GFAP*^-CKO EAE mice. **F** Quantitative analysis of the density of NeuN^+^ cells as shown in (**E**) (*n* = 5). **G** Typical images of HE staining in the lumbar spinal cords in of *SARM1*^*f/f*^ mice and *SARM1*^*GFAP*^-CKO mice, and *SARM1*^*f/f*^ EAE mice and *SARM1*^*GFAP*^-CKO EAE mice. **H** Quantitative analysis of the density of infiltrating cells as shown in (**G**) (*n* = 5). **I** The typical images of CD45^+^ immunostaining in the lumbar spinal cords of *SARM1*^*f/f*^ mice and *SARM1*^*GFAP*^-CKO mice, and *SARM1*^*f/f*^ EAE mice and *SARM1*^*GFAP*^-CKO EAE mice. **J** Quantitative analysis of the density of CD45^+^ cells as shown in (**I**) (*n* = 5). Scale bar, 50 μm. The data were mean ± SEM. Student’s *t*-test unless otherwise indicated, n.s., not significant (*p* > 0.05), **p < 0.01, ***p < 0.001.

The figure should be read:
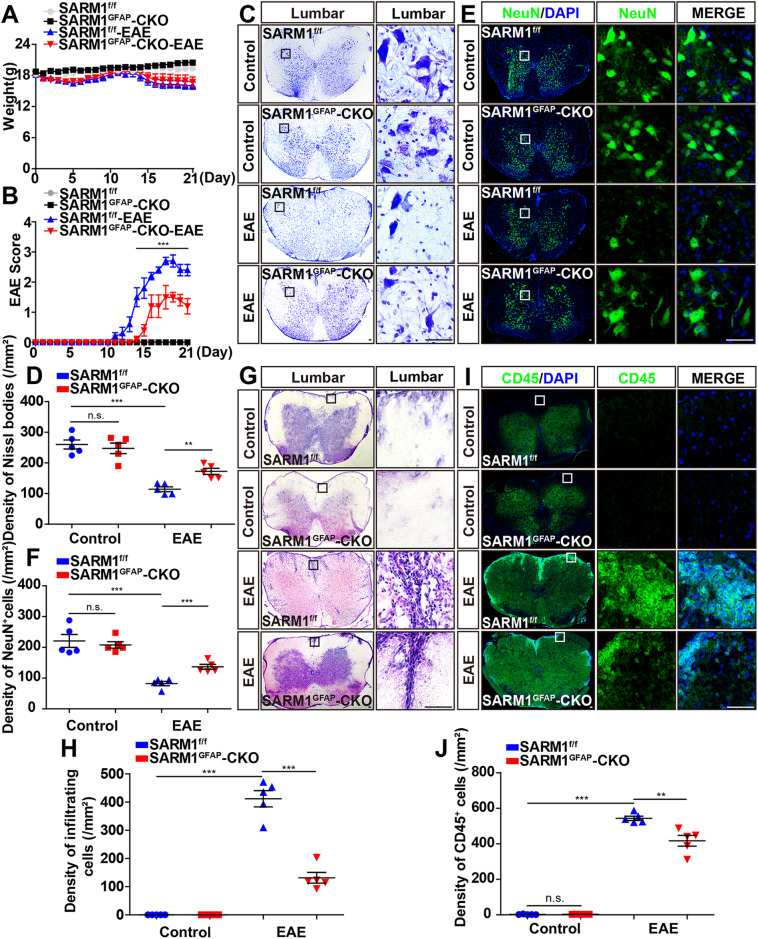


The original article has been corrected.

