# Supplementary material for: Astrocytic SARM1 promotes neuroinflammation and axonal demyelination in experimental autoimmune encephalomyelitis through inhibiting GDNF signaling
Source: Cell Death Dis. 2022 Sep 2;13(9):759. doi: 10.1038/s41419-022-05202-z (PMC9440144; doi:10.1038/s41419-022-05202-z)
Supplement: Supplementary file 2 — Supplementary material (Figure S1 Figure S2 Figure S3) [file 41419_2022_5202_MOESM2_ESM.docx]

**Supplementary material**

**
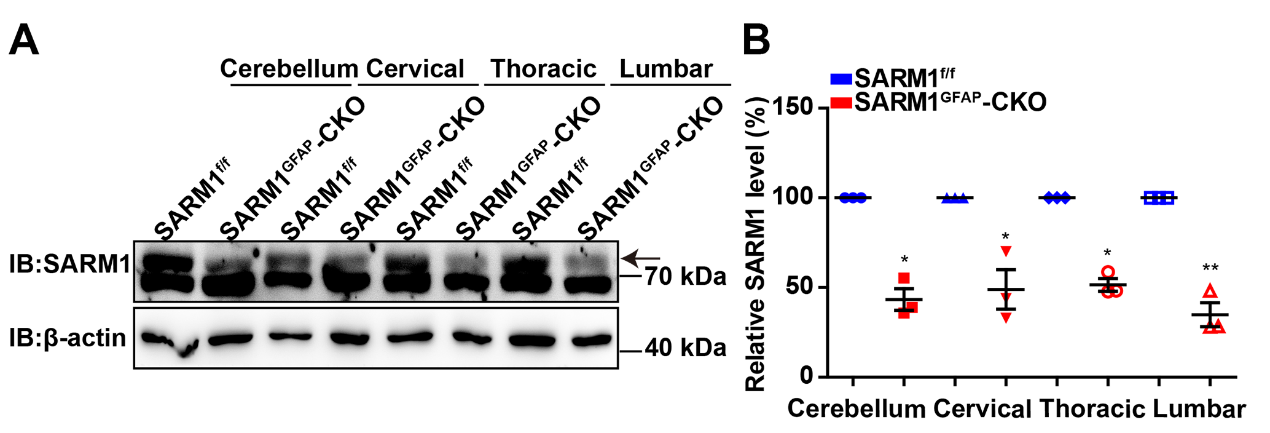
**

**Figure S1. Identification of *SARM1^GFAP^*-CKO mice.**

**(A)** Western blot detected the expression of SARM1 in cerebellum, cervical, thoracic and lumbar spinal cords of 2-month-old *SARM1^f/f^* mice and *SARM1^GFAP^*-CKO mice. **(B)** Quantification of protein levels of SARM1 as shown in (**A**) (normalized to *SARM1^f/f^* mice, n=3). Data were mean ± SEM. Student’s t-test, ^*^*p<0.05*, ^**^*p<0.01*.

**
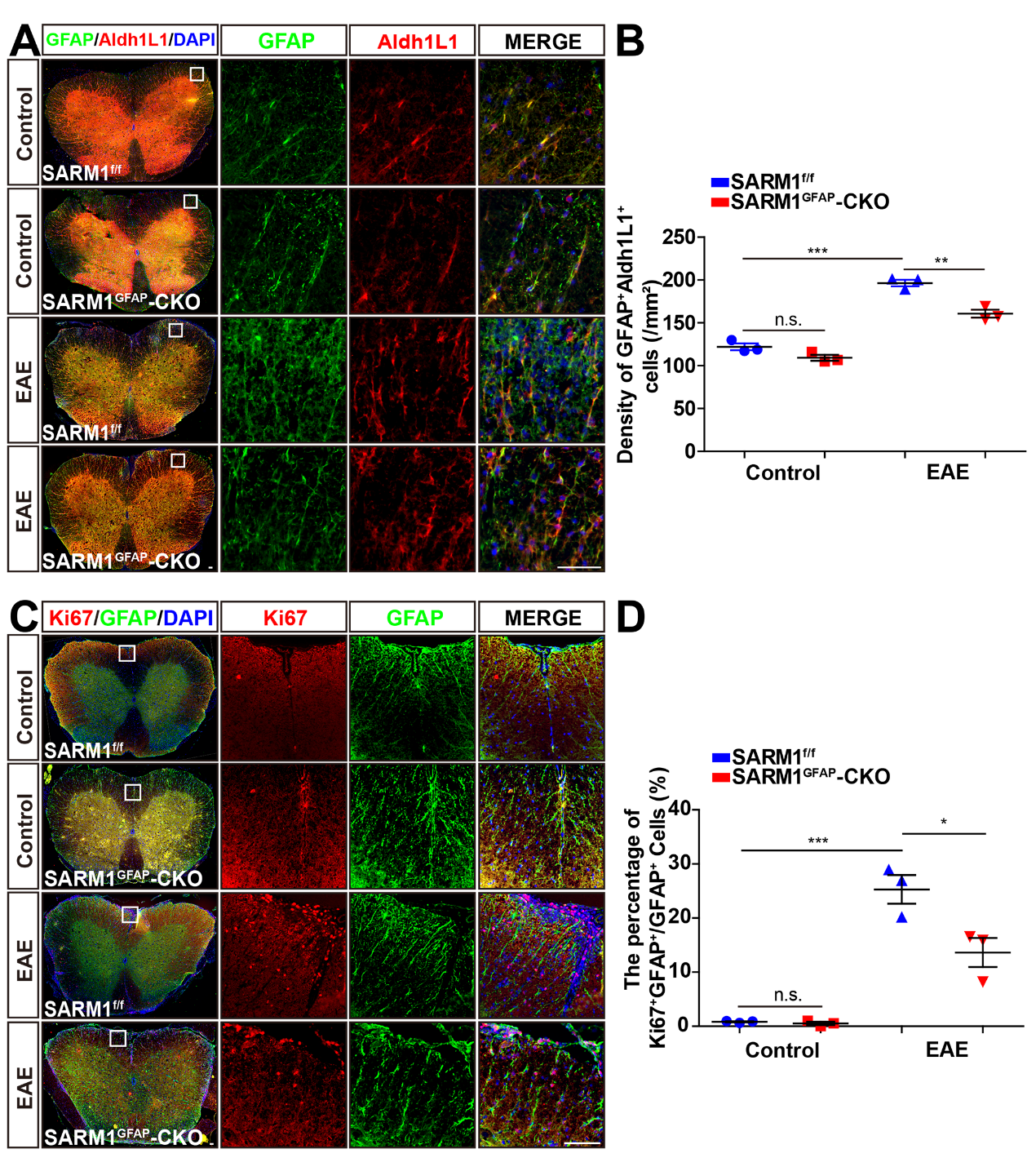
**

**Figure S2.** **The density and proliferation of astrocytes were reduced in the spinal cords of *SARM1^GFAP^*-CKO EAE mice.**

**(A)** Double immunostaining of GFAP (green) and Aldh1L1 (red) in lumbar spinal cords of *SARM1^f/f^* mice and *SARM1^GFAP^*-CKO mice, and *SARM1^f/f^* EAE mice and *SARM1^GFAP^*-CKO EAE mice. **(B)** Quantitative analysis of the density of GFAP^+^/Aldh1L1^+^ was quantified as shown in (**A**) (n=3). **(C)** Double immunostaining of Ki67 (red) and GFAP (green) in lumbar spinal cords of *SARM1^f/f^* mice and *SARM1^GFAP^*-CKO mice, and *SARM1^f/f^* EAE mice and *SARM1^GFAP^*-CKO EAE mice. **(D)** The quantitative percentage of Ki67^+^GFAP^+^ cells in the total number of GFAP^+^ cells as shown in (**C**) (n=3). Scale bar, 50 μm. Data were mean ± SEM. Student’s t-test, n.s., not significant (*p>0.05*), ^*^*p<0.05*, ^**^*p<0.01*, ^***^*p < 0.001*.

**
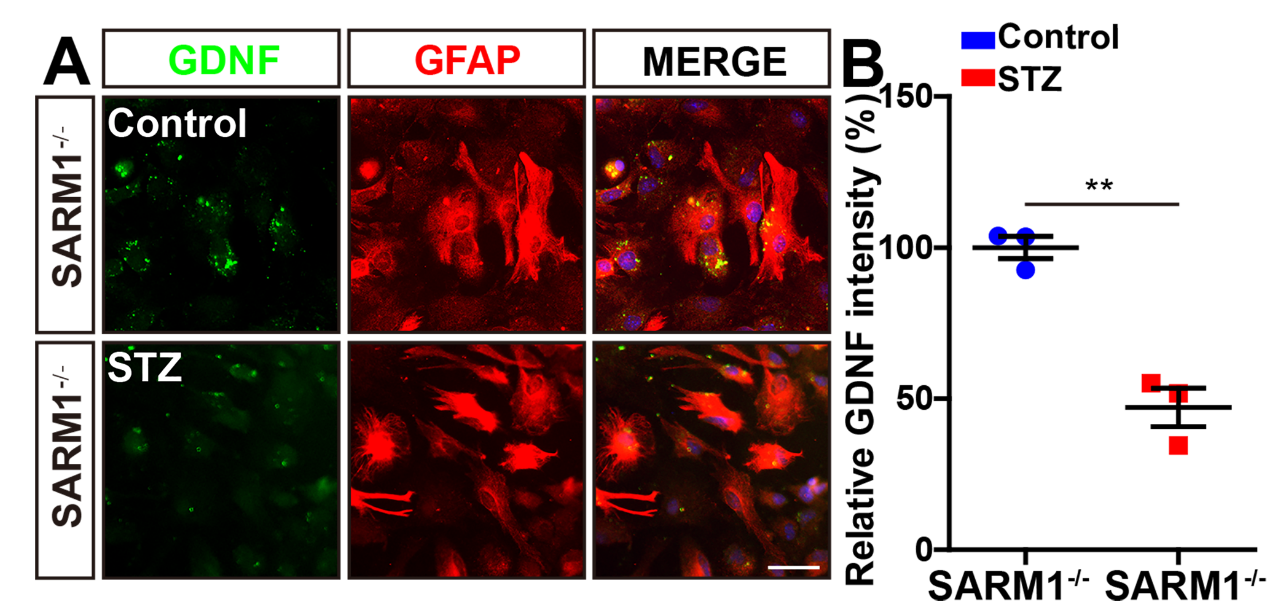
**

**Figure S3.** **GDNF was inhibited by STZ in SARM1^-/-^ astrocytes**.

**(A)** The typical images of immunostaining of GDNF (green) and GFAP (red) in SARM1^-/-^ astrocytes treated with or without STZ (100 μM). **(B)** Quantitative analysis of the intensity of GDNF was shown in (**A**) (normalized to the control group, n=3 coverslips, 15 fields/coverslip). Scale bar, 50 μm. Data were mean ± SEM. Student’s t-test, ^**^*p<0.01*.
