# Supplementary figures and images for: Astrocytic SARM1 promotes neuroinflammation and axonal demyelination in experimental autoimmune encephalomyelitis through inhibiting GDNF signaling
Source: Cell Death Dis. 2022 Sep 2;13(9):759. doi: 10.1038/s41419-022-05202-z (PMC9440144; doi:10.1038/s41419-022-05202-z)

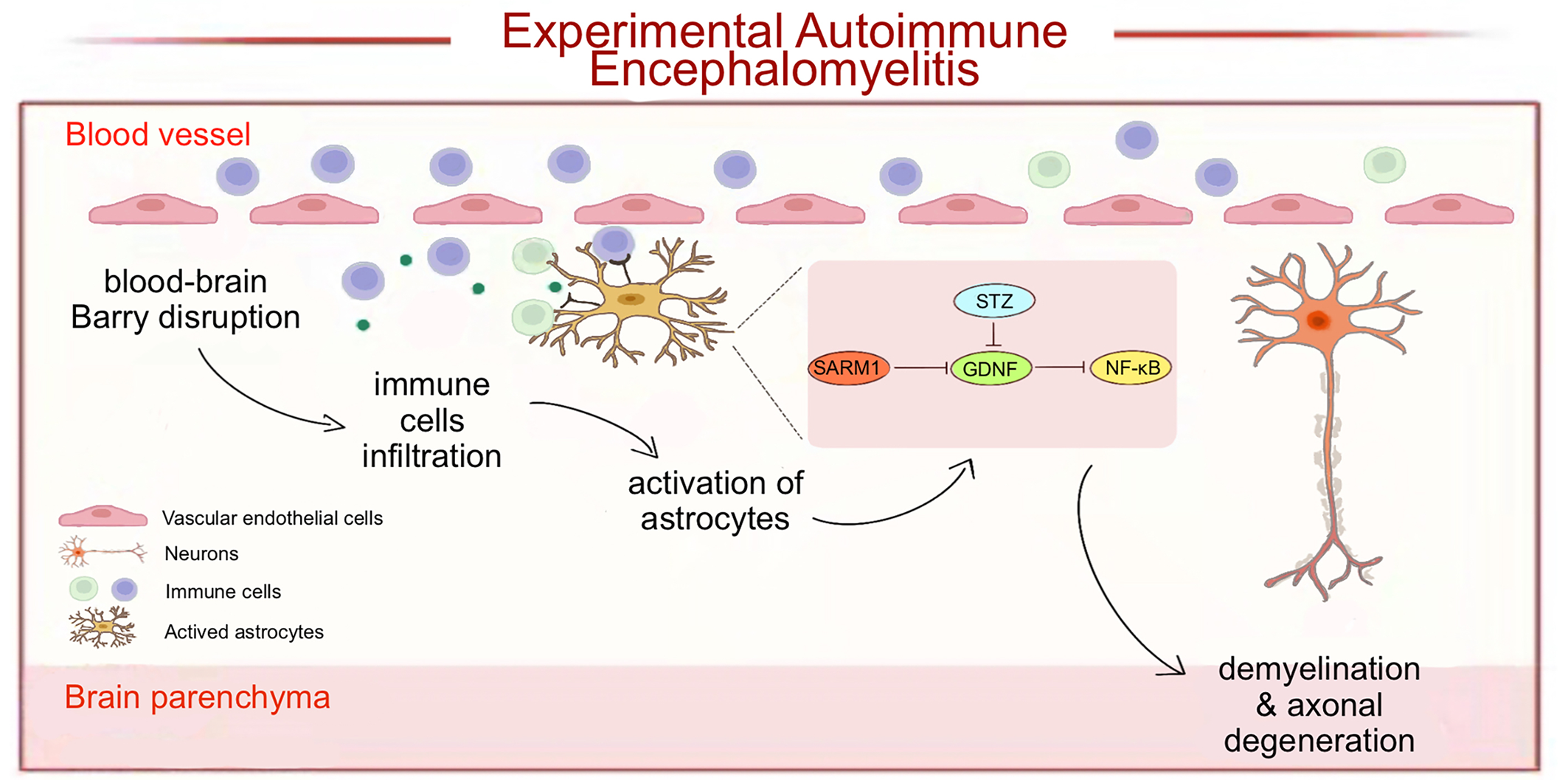

Supplement: Supplementary file 3 — Graphical Abstract [file 41419_2022_5202_MOESM3_ESM.tif]
